# Targeted interventions to prevent transitioning from acute to chronic low back pain in high-risk patients: development and delivery of a pragmatic training course of psychologically informed physical therapy for the TARGET trial

**DOI:** 10.1186/s13063-019-3350-3

**Published:** 2019-05-06

**Authors:** Jason M. Beneciuk, Steven Z. George, Carol M. Greco, Michael J. Schneider, Stephen T. Wegener, Robert B. Saper, Anthony Delitto

**Affiliations:** 1 Department of Physical Therapy, College of Public Health & Health Professions, University of Florida, Box 100154, UFHSC, Gainesville, FL 32610-0154 USA; 2 Duke Clinical Research Institute and Department of Orthopaedic Surgery, Duke University, 2400 Pratt Street, Durham, NC 27705 USA; 3 Departments of Psychiatry, Rehabilitation Science and Technology, and Physical Therapy, University of Pittsburgh School of Medicine, 3811 O’Hara Street, Pittsburgh, PA 15213 USA; 4 Department of Physical Therapy, School of Health and Rehabilitation Sciences, University of Pittsburgh, Suite 210, Bridgeside Point 1, Pittsburgh, PA 15219 USA; 5 Department of Physical Medicine and Rehab, Johns Hopkins School of Medicine, Phipps 188, 600 N. Wolfe Str., Baltimore, MD 21287 USA; 6 Boston Medical Center Department of Family Medicine, 1 Boston Medical Center Place, Dowling 5 South, Boston, MA 02118 USA; 7 School of Health and Rehabilitation Sciences, University of Pittsburgh, 4229 Forbes Tower, Pittsburgh, PA 15260 USA; 8 Brooks Rehabilitation, Clinical Research Center, 3901 University Blvd. South, Suite 103, Jacksonville, FL 32216 USA

**Keywords:** Psychologically informed physical therapy, Post-professional education, Quality improvement

## Abstract

**Background:**

Low back pain (LBP) is a public health concern because it is highly prevalent and the leading cause of disability worldwide. Psychologically informed physical therapy (PIPT) is a secondary prevention approach that first aims to identify individuals at high risk for transitioning to chronicity and then provides tailored treatment to reduce that risk. Training models that are feasible to implement with acceptable training quality are needed to improve scalability for widespread implementation of PIPT. This manuscript describes the PIPT training program that was developed for training physical therapists providing PIPT in the TARGET trial.

**Methods:**

The PIPT training program was developed, tested, and modified using an iterative process. Content development consisted of stakeholder engagement, beta testing, modification of training, and confirmation of final course objectives. Methods of delivery consisted of a website that included brief online educational modules followed by a live 8-h workshop that included video-based mock case scenarios and case-based role playing. Attitudes, beliefs, and confidence in implementing PIPT principles were assessed before and immediately after training to measure training quality and impact.

**Results:**

Early stakeholder engagement and beta testing indicated the need for increased emphasis on experiential learning opportunities and patient-centered communication training. Booster training varied extensively across TARGET sites with involvement of ‘clinician champions’ providing brief follow-up sessions identified as best practice. Favorable post-training changes in physical therapist attitudes and beliefs toward biopsychosocial treatment orientation and increased confidence in implementing PIPT principles were observed.

**Conclusions:**

PIPT training for provider participation in the TARGET trial was feasible to deliver. Course content was acceptable to physical therapists and resulted in improved beliefs and confidence in applying PIPT skills during clinical practice. Ongoing consultation and site-based continuing education were methods by which specific TARGET sites maintained or augmented PIPT skill training; however, implementing ongoing training was challenging in general. Due to the pragmatic nature of the TARGET trial, it was not possible to directly measure the effect of PIPT training on treatment fidelity, which was a limitation of our approach.

**Trial registration:**

ClinicalTrials.gov, NCT02647658. Registered on 6 January 2016.

## Background

Low back pain (LBP) is an imperative public health concern because it is highly prevalent and the leading cause of disability worldwide [1]. Global prevalence of LBP has increased by 17.3% from 2005 to 2015 and continues to be a leading cause of global years lived with disability since 1990 [1, 2]. Although most individuals will rapidly recover [3], many continue to experience ongoing or chronic pain, accounting for a large proportion of the burden associated with LBP [4, 5]. The risk factors for chronic pain are complex and multifactorial including psychological and behavioral features such as pain catastrophizing [6], fear-avoidance beliefs [7], and maladaptive pain coping [8]. These factors can be addressed via cognitive-behavioral therapy, typically offered by psychologists and other behavioral healthcare providers; however, this is seldom addressed through initial treatment delivered by primary care providers or physical therapists.

Prevention of chronic pain has emerged as a high priority in the United States, with specific emphasis placed on identifying at-risk patients and offering nonpharmacologic treatments as ‘front-line’ options [9–11]. One promising strategy is psychologically informed physical therapy (PIPT), a secondary prevention approach for LBP that first aims to identify individuals at high risk for transitioning to chronicity and then provides tailored treatment by merging impairment-focused physical therapy with cognitive behavioral therapy methods as needed to reduce that risk [12, 13]. The primary goal of PIPT is prevention of future LBP-associated disability via: 1) identification of individuals with elevated pain-associated distress and at high risk for transitioning from acute to chronic LBP; and 2) providing targeted treatment aimed at ameliorating psychological factors linked to prolonging disability in conjunction with traditional impairment-based physical therapy. PIPT optimizes variables predictive of persistent pain and is therefore consistent with a top priority of the Federal Pain Research Strategy (i.e., formalizing individualized treatment recommendations based on risk factors) [14].

Recent systematic review findings indicate psychologically based treatments can enhance physical therapy interventions for patients at high risk for prolonged LBP-related disability while emphasizing the role of risk stratification for acute LBP and specifically recommending the need for determining reproducibility in the United States and optimizing implementation in clinical practice [15, 16]. One successful risk-stratification approach for LBP uses the nine-item STarT Back Tool [17] to screen for modifiable prognostic factors, determine patient risk for developing persistent LBP-related disability, and to use that information to match patients with different care pathways. Physical therapists have played an integral role as treatment providers of PIPT in previous studies that found significant improvements in patient LBP disability and quality of life outcomes, while also resulting in less time off work and greater healthcare cost savings when compared with standard care [18, 19]. However, the training to deliver these and other psychologically based interventions has ranged from 2 to 9 days [20–25], which may not be scalable for widespread implementation in many United States healthcare systems.

There is a need for pragmatic PIPT training models that are feasible to implement with acceptable training quality and impact. These training models should also recognize clinician preferences for continuing education and be scalable to be responsive to national priorities for pain research and practice [14]. A study by Beneciuk and George [26] provides ‘proof of concept’ to support the idea that 1-day pragmatic PIPT training models are feasible and can result in improved physical therapist attitudes and beliefs about managing back pain using psychological principles. In contrast, minimal changes were observed for clinicians who did not participate in the stratified care and PIPT training module. Furthermore, therapists who received PIPT training had better 4-week patient clinical outcomes for pain intensity and self-reported disability compared with therapists who were not trained [26]. There is a timely opportunity for striking a balance between lengthy comprehensive training programs and pragmatic single-day courses. The latter are capable of providing a general overview, making such an approach feasible for widespread participation without drastically compromising training quality and impact. Such pragmatic approaches that encourage efficient treatment delivery strategies may provide a viable option for enhancing clinical practice paradigms aimed at improving patient outcomes through widespread implementation [14].

This manuscript describes the PIPT training that was developed and delivered to prepare physical therapists for providing treatment in the TARGET trial (http://www.targettrial.pitt.edu). TARGET is a large, pragmatic, cluster-randomized clinical trial of patients seen in the primary care setting with acute LBP determined by the STarT Back Tool to be at high risk for persistent LBP-related disability. In this manuscript we will first provide a brief overview of the TARGET trial design and goals to place the purpose of the training in context. Second, we will describe development and delivery of the PIPT training to prepare physical therapists at TARGET trial clinical treatment sites and describe strategies used to enhance learning. Third, we will provide data on training quality and impact. Finally, we will discuss preliminary lessons learned and provide suggestions for future pragmatically delivered PIPT training initiatives.

## Methods

### TARGET trial overview

The Targeted Interventions to Prevent Chronic Low Back Pain in High-Risk Patients (TARGET) trial is a multisite, pragmatic, cluster-randomized clinical trial studying patients with acute LBP who seek care from a primary care physician and are at high risk for persistent disability. The study is designed to assess if prompt referral of patients to physical therapists with PIPT training reduces the rate of progression to chronic LBP 6 months later (primary trial outcome) and improves back-related function as compared with guideline-based primary care management. Secondary outcomes include additional healthcare resource utilization. The TARGET trial enrolls patients from primary care clinics across multiple health systems in five geographic regions across the United States (Pittsburgh, PA; Boston, MA; Baltimore, MD; Salt Lake City, UT; and Charleston, SC) with a total planned sample size (*n* = 1860) that exceeds or is similar to previously completed studies [18, 19, 27]. The TARGET trial is funded by the Patient Centered Outcomes Research Institute and was prospectively registered with ClinicalTrials.gov (NCT02647658) on 6 January 2016. Additional trial details can be found at the ClinicalTrials.gov registry site.

### PIPT training program

**Fig. 1:**
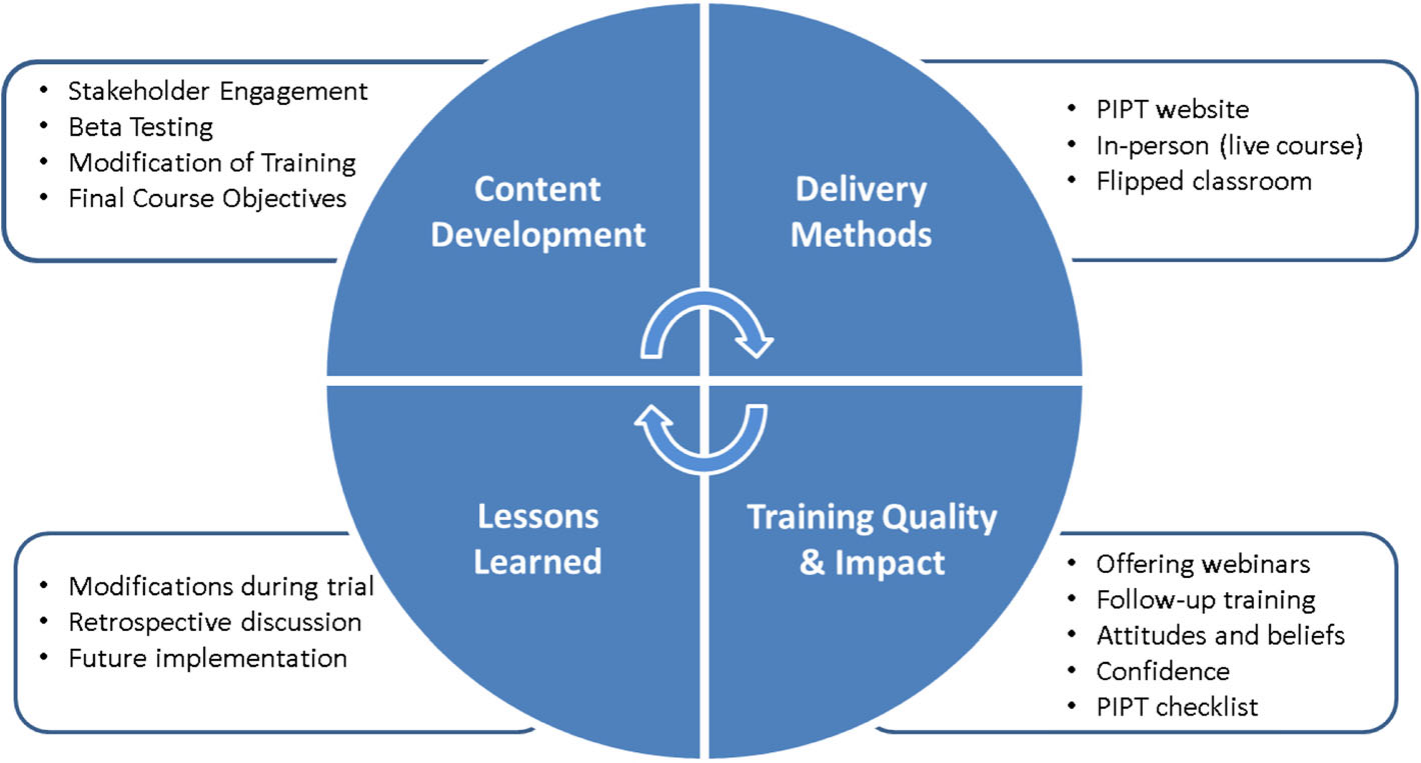
Psychologically informed physical therapy (PIPT) training program iterative process for development, testing, and modification

Considering the pragmatic study design, several factors were considered when developing the overall structure of the PIPT training program. First, there was a need to develop a multidisciplinary training team consisting of individuals representing physical therapy and clinical psychology. Second, there was the challenge of addressing the feasibility barrier of providing training to physical therapists from different healthcare systems located across five diverse geographical regions in the United States. Third, there was the importance of identifying facilitators for physical therapists to attend the PIPT training (e.g., cost, continuing education credit, and time commitment). Finally, the potential impact of discussions between physical therapists was not as concerning based on the cluster-randomized clinical trial study design, which decreased likelihood for contamination across clinical sites. Prior to trial initiation, the PIPT training program was developed, tested, and modified using an iterative process to enhance optimal effects during study training that were intended to be implemented during routine clinical practice (Fig. 1).

### Training background

Development of the PIPT training program was guided by previous protocols that have tested risk-stratification approaches for LBP using the STarT Back Tool [21, 24]. In addition, members of the TARGET intervention team (JMB and SZG) provided PIPT training for a small-scale feasibility study, training providers within a single healthcare system [26]. Key developers of the TARGET trial PIPT training program consisted of physical therapy, clinical psychology, and chiropractic providers.

### Content development

PIPT training program content development consisted of stakeholder engagement, beta testing, modification of training, and confirmation of final course objectives. Each of these stages is described in greater detail below.

#### Stakeholder engagement

Prior to providing formal PIPT training, feedback and support from key stakeholders was obtained. We initially targeted a single healthcare system (University of Pittsburgh Medical Center (UPMC), Centers for Rehab Services) to provide an introduction and overview of the TARGET trial and PIPT training program. Our initial strategy included a formal task force meeting that consisted of representation from TARGET trial investigators, healthcare system executives, outpatient clinical education, senior management, and clinicians. The key objective of this meeting was to prepare a task force of physical therapists within UPMC Centers for Rehab Services to become ‘clinical champions’ for implementing a standard biopsychosocial model for patients with musculoskeletal pain conditions.

#### Beta testing

Similar training programs have undergone beta testing to obtain critical feedback for guiding modification and prior to providing actual training in preparation for trial participation [21, 24]. Beta testing of the PIPT training program was provided for two separate cohorts of licensed physical therapists over a 2-month period in Pittsburgh (*n* = 40) and at a professional conference in National Harbor (*n* = 15). Participant feedback was collected through discussion and brief surveys, with key suggestions considered during subsequent modifications to the PIPT training program. One important outcome of the beta testing provided by course participants was the need for blended learning opportunities (i.e., strategic use of combined web-based and in-person content with interactive activities to enhance clinician learning), which led to strategies for developing additional and previously recommended [28] delivery platforms (e.g., PIPT website).

#### Modification of training

An iterative process of development for the PIPT training program was used that incorporated feedback from beta course participants, standardized self-assessments, and intervention team discussions. This led to restructuring of the live workshop to include: 1) several interactive breakout sessions, designed for the participants to gain initial experience implementing PIPT skills; 2) video-based mock clinical scenarios suitable for live course learning opportunities; 3) development of online video modules for training maintenance; and 4) increased time allotment and enhanced patient-centered communication training methods dedicated to address barriers to clinical practice integration. These modifications are consistent with enhancements provided following pilot testing of other previous training packages [24].

#### Final course objectives

Following beta testing, feedback from participants, and modification of training content and methods, final course learning objectives were finalized by the investigator team (Table 1). Collectively, the overall objectives of the PIPT training program were to provide participants with a summary of evidence and clinical skills necessary to support implementing PIPT principles into routine clinical practice for patients identified as being at high risk for transitioning from acute to chronic LBP. Methods of delivery (described below) were intended to promote a blended learning (i.e., flipped classroom) environment with instructional strategies guided by specific learning objectives [29, 30]. Flipped classroom pedagogy principles included as part of the PIPT training program included flexible learning, improved preparation for the live workshop, self-reflection, peer-learning, and enhanced rapport with instructors.

**Table 1:** Psychologically Informed Physical Therapy Training Course Learning Objectives.

1.	Summarize relationships between pain neuroscience, pain models, and the development and maintenance of chronic low back pain.
2.	Identify patients at high risk for transitioning from acute to chronic low back pain.
3.	Apply targeted treatment for patients at high risk for transitioning from acute to chronic low back pain.
4.	Understand primary assumptions of CBT and specific skills associated with CBT based interventions.
5.	Recognize effective communication skills and be able to implement as a key component to PIPT.
6.	Differentiate key principles and application between graded activity and graded exposure.
7.	Review the Low Back Pain Clinical Practice Guidelines from the Orthopaedic Section of the American Physical Therapy Association to become familiar with: 1) ICF-based classifications; 2) symptoms; 3) impairments; and 4) suggested intervention strategies.
8.	Be able to implement PIPT practice principles for patients with low back pain.

*PIPT* Psychologically Informed Physical Therapy, *CBT* Cognitive-Behavioral Therapy, *ICF* International Classification of Functioning, Disability, and Health

#### Final course content

**Fig. 2:**
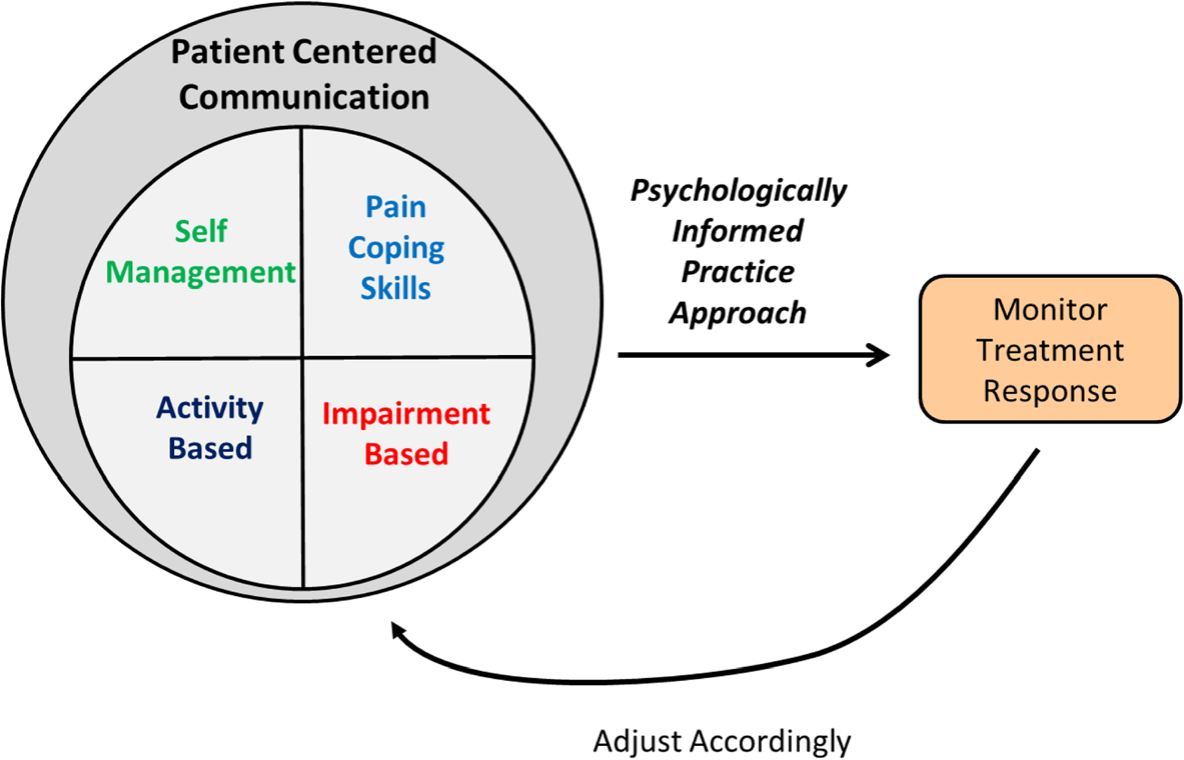
Overview of key psychologically informed physical therapy (PIPT) treatment components for high-risk patients in the TARGET trial

A brief description of the final course content is provided in Table 2 with greater details in the Appendix. Course content was broadly described as either ‘Overview’ (providing rationale and supporting data for the PIPT approach) or ‘PIPT Management’ (providing specific principles or skills with demonstration and practice). ‘PIPT Management’ content consisted of: 1) patient-centered communication; 2) pain coping skills; 3) patient education; 4) activity-based intervention; 5) impairment-based intervention; and 6) treatment monitoring components (Fig. 2). The course content was provided in sequential order for all training sessions (Appendix).

**Table 2:** Psychologically informed physical therapy (PIPT) training course content and methods of delivery

	Approximate time allotment dedicated during live workshop	Methods of delivery		
		PowerPoint presentations and instructor-led group discussion	Video-based mock case scenarios	Case-based role playing (breakout sessions)
Overview				
Pain science update	30 min	X		
PIPT overview	30 min	X		
Risk stratification		X		
Targeted treatment		X		
Cognitive behavioral therapy	30 min	X		
Self-reflection	45 min	X	X	X
PIPT management				
Patient-centered communication	1 h, 45 min			
Active listening		X	X	
Motivational interviewing		X	X	X
Goal-setting		X	X	
Pain coping skills	1 h, 15 min			
Physiologic relaxation		X		X
Imagery		X		X
Replacing cognitive distortions		X	X	X
Patient education	15 min	X		
Activity-based	60 min			
Graded exercise		X	X	X
Graded exposure		X	X	X
Impairment-based	30 min			
Clinical practice guidelines		X		
Treatment monitoring	30 min	X		
Challenges and opportunities	30 min	X	X

### Methods of delivery

Consistent with the pragmatic study design of the TARGET trial, the training was designed to be easily replicated in clinical settings to augment delivery of PIPT implementation. Flipped classroom instructional methods were integrated to enhance preparation for the live workshop, with the intention that each site would determine specific methods favorable for that specific geographical region and health delivery system.

#### PIPT website

The TARGET trial website (http://www.targettrial.pitt.edu/) provided an alternative delivery platform for content resources that included an overview of the TARGET trial and provider resources (including key recommended readings). Course participants registered for formal training courses were directed to a separate PIPT website that provided a course overview, learning objectives, education modules, and additional educational resources for patients. Twelve brief pre-course online educational modules were developed to provide necessary foundational information required to optimize the experiential nature of the 1-day live workshop and promote a flipped classroom learning model. These online educational modules were designed to be viewed in sequence, with each module ranging from 8 to 22 min in duration (total viewing time, 150 min). Links to voiceover PowerPoint presentations, electronic handouts, and audio files were provided for each module to offer course participants different learning platform options. Physical therapists had the opportunity to obtain 2.5 h of continuing education credit after viewing all the online modules. Viewing online video modules was highly recommended prior to attending the live workshop; however, we did not monitor everyone who accessed the website. Physical therapists seeking continuing education credit were required to complete a quiz after each online module, thereby providing a method to monitor online attendance certificate of completion eligibility. In situations where physical therapists were not seeking continuing education credit (as not required in all states), quizzes were not administered and there was no method to objectively monitor online attendance.

#### Live workshop

All sites participating in the TARGET trial had practicing physical therapists who would deliver PIPT at a local clinic. The sites were required to host live workshops as part of their site participation requirement. However, our ability to monitor which specific physical therapists attended and where they were practicing in a given site was limited as registration processes varied across TARGET site locations. Live 1-day workshops were provided by at least one physical therapist and clinical psychologist up to three times at each of the five TARGET sites throughout the United States (Pittsburgh, PA; Boston, MA; Baltimore, MD; Salt Lake City, UT; and Charleston, SC). Combinations of teaching methods (Table 2) were used during each 8-h workshop, including: PowerPoint presentations; video-based mock case scenarios depicting appropriate and inappropriate communication styles; and case-based role playing.

To enhance clinical skills training, we utilized several structured teaching and learning strategies, including: 1) instructor-led teaching on specific clinical skills; 2) instructor-led, case-based role playing with mock patient; 3) course participant-led, case-based role playing (i.e., breakout sessions) where smaller groups of two to four participants each assumed different stakeholder roles (e.g., patient, clinician, and observer) for a variety of clinical scenarios; and 4) class discussion to provide individual learning experience perspectives. To further enhance learning, participants were encouraged to demonstrate newly acquired clinical skills that were evaluated by instructors and other participants for real-time feedback. This case-based role playing was used to develop clinical skills involving self-reflection, motivational interviewing, pain coping skills, and activity-based interventions. Barriers and facilitators to implementing PIPT components (Fig. 2) during routine clinical practice were addressed throughout the live workshops.

#### PIPT course materials

Each physical therapist was provided with course materials at the live 1-day workshop that could be referenced afterwards. These materials consisted of workshop content, including specific descriptions and scenarios pertaining to PIPT interventions such as patient-centered communication, pain coping skills, patient education, activity-based intervention, impairment-based intervention, and treatment monitoring components.

### Quality improvement strategies to enhance and assess quality and impact of provider training

Establishing treatment fidelity to ensure the reliability and validity of behavioral interventions has been identified as a major challenge [31, 32]. The National Institutes of Health Behavior Change Consortium (NIHBCC) has developed and recently updated a treatment fidelity framework consisting of five domains (i.e., study design, training of providers, treatment delivery, treatment receipt, and treatment enactment) [31, 32]. Consistent with the pragmatic nature of the TARGET trial, a balance between feasibility and obtaining comprehensive fidelity assessments was considered [33]. The PIPT program was designed to promote treatment fidelity by providing quality training that impacted key provider factors and that could be replicated. Thus, we incorporated quality improvement strategies (PIPT treatment checklist and booster training) and measures (physical therapist attitudes, beliefs and confidence, described in greater detail below) to enhance treatment quality and the impact of training.

#### Strategies to enhance treatment quality

##### PIPT treatment checklist

To promote treatment fidelity, physical therapists were trained to indicate specific PIPT treatment content delivered during patient care by completing self-report checklists [31–34]. Strategies for administering checklists varied across geographical regions, ranging from traditional hardcopy methods to direct entry into the electronic medical record.

##### Booster training

Due to the pragmatic nature of the trial, the amount and frequency of follow-up communication and training maintenance was different in each geographical region. As a result, booster or refresher training varied extensively, with course instructors and site coordinators offering several options following the live workshop. All course participants were provided with options for continued remote communication with instructors, and were encouraged to submit follow-up questions and testimonials to promote a flipped classroom learning environment. One implementation process that may be described as possible ‘best practice’ within this trial consisted of 1-hour follow-up sessions provided at several clinical sites in the Salt Lake City, UT, region that were focused on improving specific PIPT skills that physical therapists found difficult to implement. For example, prior to the course, participants indicated difficulty with initiating PIPT interventions, specifically related to patient-provider communication. Cognitive reassurance (engaging the patient in education) was thoroughly discussed in group settings and motivational interviewing strategies were revisited through case-based role playing using specific patient scenarios that were led by site mentors. Another strategy in Pittsburgh, PA, Baltimore, MD, and Boston, MA, regions consisted of offering brief 45- to 60-min webinars where course participants were asked to submit topical questions, with instructors and clinical champions providing strategies to overcome barriers to successful implementation.

#### Measures to assess training impact

Physical therapists that attended the live workshop were administered questionnaires to assess attitudes, beliefs, and confidence (described below). Course instructors did not provide any instruction or advice for how to respond to individual questionnaire items.

##### Attitudes and beliefs

Physical therapist attitudes and beliefs about biomedical and biopsychosocial treatment orientations were assessed before training, immediately after completion of training, and 4 months later using the Pain Attitudes and Beliefs Scale for Physical Therapists (PABS-PT) [35, 36]. The PABS-PT consists of 19 items about treatment orientation that are rated using a six-point Likert scale ranging from “totally disagree” to “totally agree”. The PABS-PT biomedical scale (10 items) has a potential score range from 10 to 60, and the PABS-PT biopsychosocial scale (9 items) has a potential score range from 9 to 54, with higher scores indicating increased biomedical or biopsychosocial treatment orientation depending upon the respective scale. TARGET site leaders initiated a request for follow-up assessment 4 months after training through email that directed course attendees to a remote website containing an electronic version of the PABS-PT with reminders being sent 2 weeks later.

##### Confidence in PIPT skill application

Physical therapist confidence in implementing PIPT principles was assessed before training and upon completion of training (same day). Specifically, participants were asked to “rate your level of confidence with implementing psychological informed principles during clinical practice” using an 11-point scale (range 0 to 10) with 0 indicating “no confidence” and 10 indicating “extreme confidence”.

#### Quality improvement evaluation of PIPT training program

A total of 471 outpatient physical therapists attended at least one live workshop and completed pre-training questionnaires. Means and standard deviations (SDs) were calculated for available continuous variables (i.e., age, years in practice, PABS-PT, and confidence scores) for the entire study sample by each TARGET site (Table 3). Paired-samples *t* tests were used to assess for pre- to post-course changes in the scores derived from the PABS-PT (biomedical and behavioral scale) and confidence in applying PIPT questionnaires. Three separate multiple regression models were then used to explore the contribution of TARGET site location and viewing pre-course online video modules as predictors of post-course scores. For each model, Block 1 accounted for pre-training PABS-PT or confidence scores depending upon the outcome of interest; Block 2 added TARGET site location to Block 1; and Block 3 added self-report response to the question about viewing pre-course video modules (Yes or No) to Block 2. Finally, one-way analysis of variance with Bonferroni post-hoc procedures was used to compare physical therapist PABS-PT (biomedical and behavioral scale) and confidence residualized change scores between TARGET site locations to evaluate for training replicability.

**Table 3:** Psychologically informed physical therapy (PIPT) course participant characteristics (*n* = 471)

	Total sample (*n* = 471)	Pittsburgh, PA (*n* = 77)	Boston, MA (*n* = 61)	Salt Lake City, UT (*n* = 80)	Baltimore, MD (*n* = 111)	Charleston, SC (*n* = 142)	*P* Value*
Age (years)	38.1 (11.0)	40.3 (11.2)	32.4 (8.1)	39.3 (10.3)	36.9 (11.2)	39.5 (11.4)	< 0.001
Years in practice	11.4 (10.6)	14.6 (11.5)	6.1 (7.0)	11.0 (10.4)	10.9 (11.4)	12.6 (10.2)	< 0.001
PABS-PT biomedical (pre-training)	31.0 (6.8)	30.3 (6.5)	30.4 (7.6)	28.2 (6.8)	31.0 (6.8)	33.2 (6.0)	< 0.001
PABS-PT biomedical (post-training)	25.2 (7.2)	26.0 (7.0)	24.3 (7.6)	23.1 (6.8)	25.2 (7.1)	26.2 (7.4)	0.032
PABS-PT behavioral (pre-training)	36.9 (3.7)	36.6 (3.2)	36.9 (4.2)	38.3 (3.8)	37.1 (3.3)	36.1 (3.7)	0.001
PABS-PT behavioral (post-training)	41.3 (4.2)	40.1 (3.8)	41.7 (5.2)	41.7 (4.3)	41.7 (4.4)	41.2 (3.5)	0.067
Confidence (pre-training)	4.8 (2.2)	4.8 (2.1)	4.6 (2.3)	4.9 (2.2)	5.1 (2.3)	4.5 (2.2)	0.292
Confidence (post-training)	7.3 (1.9)	7.0 (1.7)	5.6 (3.1)	7.4 (1.8)	7.8 (1.2)	7.7 (1.4)	< 0.001

Results are shown as mean (standard deviation)

*PABS-PT* Pain Attitudes and Beliefs Scale for Physical Therapists

*One-way analysis of variance to compare between TARGET site locations

## Results

### Attitudes and beliefs

Follow-up assessments upon completion of training were performed for 91.5% (431/471) of course participants. PABS-PT biomedical scale scores decreased from 31.1 (SD = 6.8) to 25.0 (SD = 7.1) (*P* < 0.001), and behavioral scale scores increased from 36.8 (SD = 4.8) to 41.4 (SD = 5.2) (*P* < 0.001). Regression models explained 38% and 17% of the variance in post-course PABST-PT biomedical and behavioral scale scores, respectively. Pre-course PABS-PT biomedical (β = 0.62, *P* < 0.001) and behavioral (β = 0.41, *P* < 0.001) scale scores were the strongest predictors of post-course PABS-PT biomedical and behavioral scale scores, respectively. TARGET site location only added 1% additional variability to prediction of post-course PABS-PT biomedical (β = 0.09, *P* = 0.022) and behavioral (β = 0.12, *P* = 0.018) scale scores. Viewing pre-course online modules did not significantly explain any additional variability in post-course PABS-PT biomedical or behavioral scale scores (*P* > 0.05). After adjustment for pre-course scores by each site location, there were no differences in PABS-PT biomedical (*P* = 0.140) or behavioral (*P* = 0.095) scale change scores between TARGET site locations. A total of 134 (28.4%) course participants completed a web-based version of the PABS-PT 4 months after training with biomedical (25.4 ± 7.9) and behavioral (40.9 ± 4.4) scale scores observed; however, these data were de-identified which does not allow us to determine if sustained scores were maintained over a longer duration of time.

### Confidence in PIPT skill application

Follow-up assessments of confidence upon completion of training (same day) were performed for 96.2% (453/471) of course participants. We were not able to capture any additional assessments of confidence at 4 months. Confidence in the ability to implement PIPT principles increased from 4.8 (SD = 2.2) to 7.3 (SD = 1.9) (*P* < 0.001). The regression model explained 27% of the variance in post-course confidence scores. Pre-course confidence score (β = 0.46, *P* < 0.001) was the strongest predictor explaining 20% of the variance in post-course confidence TARGET site location (β = 0.21, *P* < 0.001) added 5% additional variability to the prediction of post-course confidence scores. Viewing pre-course online modules (β = 0.12, *P* = 0.003) explained 2% additional variability in post-course confidence scores. Greater post-course confidence scores were observed for those who viewed modules when compared with those who did not (mean = 7.7 (SD = 1.3) versus 7.2 (SD = 1.7), *P* = 0.004). After adjustment for pre-course scores, differences in confidence change scores between TARGET site locations were observed (*P* < 0.001). Specifically, Boston, MA, reported less improvement in confidence compared with Pittsburgh, PA (mean difference = 1.2, 95% confidence interval (CI) = 0.1–2.3, *P* = 0.029), Salt Lake City, UT (mean difference = 1.6, 95% CI = 0.5–2.7, *P* = 0.001), Baltimore, MD (mean difference = 1.7, 95% CI = 0.6–2.7, *P* < 0.001), and Charleston, SC (mean difference = 2.4, 95% CI = 1.3–3.4, *P* < 0.001). Additional findings indicated that Boston, MA, participants were younger in age (32.4 years (SD = 8.1)) and had less experience in clinical practice [6.1 years (SD = 7.0)) compared with all other TARGET site locations (*P* < 0.001), which may have influenced observed changes in confidence (Table 3).

## Discussion

The overall objectives of the training course were to provide physical therapists with a summary of evidence and clinical skills necessary to support the implementation of PIPT principles into clinical practice for patients identified as being at high risk for transitioning from acute to chronic LBP. Our experiences have provided several important “lessons learned” that can be used to guide future study of PIPT implementation for long-term sustainability [37].

### Emphasis on experiential learning

During beta testing, course participants provided consistent feedback about the need to reduce didactics and increase the amount of time devoted to experiential learning experiences. Therefore, PIPT treatment concepts were introduced using video and live mock case scenarios that transitioned into small group practice sessions during each 8-h workshop. These teaching principles were also utilized in certain regions following initial training as a component of booster training and could perhaps be described as ‘best practice’ within the TARGET trial.

### PIPT clinician champions

Several strategies to enhance routine application of PIPT principles following the live course and during active patient enrollment periods (i.e., booster training) were planned during program development stages; however, due to the pragmatic nature of the trial and geographical distribution of health systems, implementing these efforts was associated with considerable heterogeneity. For example, clinician-generated case reports that were intended to be the focus of dynamic learning communities were conceptualized as being a virtuous strategy. However, engaging clinicians to be accountable for active learning initiatives was a difficult process and only resulted in a small number of case examples (potentially due to busy, high-volume clinical practices). Therefore, future implementation efforts should focus on strategies to identify and incentivize clinical champions within a health system or small region for leading subsequent active learning initiatives (e.g., webinars, formal mentoring opportunities) following initial training. Optimally, these individuals should demonstrate special interest and skill in PIPT and could be valuable resources for circumstances where continued on-site interaction with primary trainers is not feasible**.** Personal communication with physical therapists that received PIPT training for a previous smaller scale study and provided similar treatment indicated the need for additional follow-up opportunities to address barriers in clinical practice following the training course [26].

### Need for specialized training

Despite recent recommendations for increased delivery of psychological-based treatment [9, 10] and enthusiasm for risk-stratification approaches to LBP management [14, 15] there are challenges to successful widespread implementation. For example, a potent barrier to successfully delivering psychological-based treatment is the vital need for additional specific post-professional training [12, 38–41]. This dilemma is particularly relevant to healthcare providers where biomedical or impairment-based perspectives have been predominantly emphasized during entry-level education and clinical practice, thereby resulting in clinicians who are not confident or who are unprepared for delivering psychological-based interventions [38, 39, 42]. For example, our findings indicated that fewer years in clinical practice was associated with less improvement in confidence after attending the live PIPT workshop, potentially suggesting that less-experienced physical therapists (i.e., new graduates) may not be adequately prepared to successfully implement PIPT strategies with patients or who require additional training. Consequently, gaining additional specific knowledge, problem-solving skills, and practical experience through formal mentoring opportunities that incorporate booster training is a vital necessity for beneficial shifts in clinical practice paradigms to occur. However, many PIPT or cognitive behavioral treatment approaches require specialized time-intensive training, which may not be feasible for all clinicians and may perhaps present a significant barrier to successful widespread future clinical implementation. Providing single-day overview courses that are followed by structured mentorship experiences over an extended period of time may perhaps provide a viable model for future PIPT training programs.

### Patient-centered communication

In our experience, the initial perception of physical therapists was that implementing PIPT strategies in practice would be challenging. Training in patient-centered communication appears to be an important component for integrating PIPT into routine clinical practice. As previously indicated, increased time and enhanced training methods were dedicated to patient-centered communication during the preliminary phases of our training program based on early feedback from participants involving barriers to clinical practice integration. Similar challenges and enhancements to communication content and delivery methods have been acknowledged during development of other PIPT-based training programs [24, 43]. Clinician challenges to providing patient-centered and biopsychosocial-oriented communication for patients with LBP is common [38–40, 42, 44], which is not surprising considering the lack of content dedicated to this topic during entry-level physical therapy training programs. Specifically, our patient-centered communication interventions were enhanced early during trial training stages by integrating motivational interviewing skill development with significant time permitted for: 1) instructor-led, case-based role playing with mock patients; 2) course participant-led, case-based role playing (i.e., breakout sessions) where smaller groups of two to four participants each assumed different stakeholder roles (e.g., patient, clinician, and observer) for a variety of clinical scenarios; and 3) class discussion to provide individual learning experience perspectives. Future implementation strategies should strongly consider providing direct examples that combine patient-centered communication skills and other PIPT interventions such as graded activity or graded exposure to optimize treatment efficiency. This approach may be particularly beneficial as most patient expectations for physical therapy treatment may not be aligned with PIPT-based principles.

### Provider training

Training quality and impact assessment could have been further enhanced by including formal assessment of skill acquisition, standardized methods to prevent skills drift, and providing accommodations to diverse learning styles [32]. Formal assessment to determine if learning objectives were attained would have also potentially strengthened the likelihood of subsequent PIPT implementation during trial participation; however, this additional assessment may have also reduced the pragmatic nature of the training program and limited physical therapist engagement. PIPT training program evaluation was not the primary aim of the TARGET trial, and was completed under the auspices of quality improvement, not educational research. Therefore, we were limited on the amount of data collected from physical therapists who attended the live workshop and the ability to conduct formal outcome assessments.

In our opinion, the PABS-PT and confidence measures provided one viable option for assessment of the impact of PIPT training on clinician attitudes and beliefs. Updated strategies for assessment provided by the NIHBCC have indicated the importance of ensuring “buy-in” for treatment [32], with previous studies having considered clinician attitudes and beliefs to guide development of targeted treatment and training packages [21]. We observed favorable treatment orientation shifts from predominantly biomedical to biopsychosocial following attendance at the live workshop, which is consistent with previous studies where a similar duration of PIPT training was delivered [26, 45]. Our viewpoint is that this favorable change in treatment orientation and confidence is a practical indicator that the PIPT training has potential for altering the attitudes, beliefs, and confidence of therapists. However, we acknowledge that additional assessment through direct observation or formal assessment of competency (which may not be feasible in clinical settings) would be needed to determine if the PIPT training resulted in behavioral change for the provider and if these changes are associated with improvements in patient outcomes.

These findings highlight the need to consider the attitudes and beliefs of clinicians regarding treatment orientation when introducing new treatment approaches, such as PIPT, if they are to be adopted in clinical practice [46]. Systematic review findings indicate that healthcare providers with predominantly biomedical treatment orientations are more likely to suggest limited work and physical activity, and are less likely to adhere to clinical practice guidelines that emphasize activation for patients with LBP [46, 47]. Changing provider beliefs is critical if we are to optimize care for patients who widely believe their persistent LBP results from anatomical or biomechanical causes [48]. Therefore, future studies should consider long-term assessment of PIPT training quality and impact to determine if favorable changes in the attitudes and beliefs of clinicians are sustained over time and how, or if, these changes influence patient outcomes.

### Long-term follow-up to sustain changes

Considering that our strategies for continual engagement with physical therapists following in-person training may have been less than optimal, we suggest several strategies to enhance this process in an effort to sustain beneficial changes in attitudes and beliefs over long-term periods. First, sustained communication between instructors, site leaders, and course participants may enhance PIPT training maintenance opportunities. Second, continuing education credit or organizational quality improvement initiatives may provide clinicians with incentive for participating in maintenance activities. Third, system-level recognition for cohorts achieving specific maintenance participation rates may provide clinicians with a sense of personal satisfaction. Finally, we used the PABS-PT to assess clinician attitudes and beliefs about treatment orientations and a single question to assess confidence in implementing PIPT principles; therefore, ongoing assessment and feedback may assist with skill maintenance.

### Suggestions to increase scalability

Based on our experiences, future pragmatically delivered PIPT training initiatives should consider providing single-day overview courses that are followed by structured mentorship experiences (i.e., either remotely online or in-person) over an extended period of time. This may provide a viable continuing education model for future PIPT training programs that can be led by local clinical champions, moderated by course instructors, and provided using remote learning platforms (e.g., webinars, discussion boards). Based on our findings, we also suggest that future PIPT training programs be tailored to participant characteristics and clinical experience. Specifically, content knowledge, attitudes, beliefs, and confidence need to be considered as course content, and delivery methods may need to be modified. Providing clinicians with incentives to assume these leadership roles will be important since participation and time spent developing formal case reports for ‘real-world’ learning experiences (as an example) will require personal commitment and, most likely, dedication of time outside of clinical practice.

## Conclusions

The PIPT training in the TARGET trial, which consisted of online educational modules followed by a 1-day live discussion and skills-based training, was feasible to deliver as part of a large, pragmatic trial. The final format for the course was acceptable to physical therapists and resulted in improvement in biopsychosocial attitudes and beliefs and confidence in applying PIPT skills during clinical practice. Ongoing consultation and site-based continuing education were methods by which specific TARGET sites maintained or augmented the PIPT skill training; however, ongoing training was challenging for most TARGET sites in general. Treatment fidelity was not measured directly, which was a limitation to our training approach and will continue to be a struggle for future pragmatic trials that are charged with delivering interventions as part of routine clinical practice.

## Appendix

**Table 4:** Psychologically informed physical therapy (PIPT) training course content details

	Content description
Overview	
Pain science update	Variability as an inherent feature of the pain experience; psychological factors as indicators to provide explanation of pain-related patient differences
PIPT overview	Identification of pain-associated psychological distress and use of targeted treatment approaches as key tenets of PIPT; preventing transition to chronic back pain is a primary outcome goal for PIPT
Risk stratification	Patient subgrouping; prognostic risk stratification; STarT Back Screening Tool
Targeted treatment	General overview of recommended treatment pathways for STarT Back low, medium, and high risk
Cognitive behavioral therapy	Principal CBT assumptions (e.g., treatment to address cognitive, emotional, and behavioral dimensions); CBT components (e.g., goal setting, skill development, monitoring, maintenance); distinction between CBT and PIPT
Self-reflection	Reflection on working with patient who is challenging to therapist (recognizing own thoughts, moods, sensations); empowerment through awareness; challenges and opportunities for physical therapist
PIPT management	
Patient-centered communication	
Active listening	Examples, roadblocks, strategies
Motivational interviewing	Components (acceptance, compassion, evocation, partnership); strategies (open-ended questions, affirmations, reflections, summary)
Goal-setting	Collaborative process; assessing patient confidence, commitment, and barriers; SMART goals (specific, measurable, attainable, relevant, time-based)
Pain coping skills	
Physiologic relaxation	Diaphragmatic breathing methods, progressive muscle relaxation
Imagery	Pleasant place imagery
Replacing cognitive distortions	Using STarT Back Tool responses to identify and replace unhelpful thinking; treatment time efficiency
Patient education	Interactive, online program to help individuals manage pain following an injury
Activity-based	
Graded exercise	Operant-conditioning model; quota-based dosage system; progression; reward strategies
Graded exposure	Phobia model; hierarchical exposure approach; progression based on decreased fear of activity
Impairment-based	
Clinical practice guidelines	Review of orthopedic section of the APTA Low Back Pain Clinical Practice Guidelines
Treatment monitoring	Treatment monitoring concept and suggested methods
Challenges and opportunities	Open discussion with reference to peer-reviewed literature surrounding topic

*APTA* American Physical Therapy Association, *CBT* cognitive behavioral therapy
